# Increased Nonconducted P-Wave Arrhythmias after a Single Oil Fly Ash Inhalation Exposure in Hypertensive Rats

**DOI:** 10.1289/ehp.0800129

**Published:** 2008-12-31

**Authors:** Aimen K. Farraj, Najwa Haykal-Coates, Darrell W. Winsett, Mehdi S. Hazari, Alex P. Carll, William H. Rowan, Allen D. Ledbetter, Wayne E. Cascio, Daniel L. Costa

**Affiliations:** 1 Experimental Toxicology Division, National Health and Environmental Effects Research Laboratory, U.S. Environmental Protection Agency, Research Triangle Park, North Carolina, USA;; 2 Environmental Sciences and Engineering, University of North Carolina at Chapel Hill, Chapel Hill, North Carolina, USA;; 3 ITT Corporation, Advanced Engineering and Sciences, Alexandria, Virginia, USA;; 4 Brody School of Medicine, East Carolina University, Greenville, North Carolina, USA;; 5 Office of Research and Development, U.S. Environmental Protection Agency, Research Triangle Park, North Carolina, USA

**Keywords:** arrhythmia, electrocardiogram, heart rate variability, hypertension, inhalation, nonconducted P-waves, particulate matter, rats, residual oil fly ash

## Abstract

**Background:**

Exposure to combustion-derived fine particulate matter (PM) is associated with increased cardiovascular morbidity and mortality especially in individuals with cardiovascular disease, including hypertension. PM inhalation causes several adverse changes in cardiac function that are reflected in the electrocardiogram (ECG), including altered cardiac rhythm, myocardial ischemia, and reduced heart rate variability (HRV). The sensitivity and reliability of ECG-derived parameters as indicators of the cardiovascular toxicity of PM in rats are unclear.

**Objective:**

We hypothesized that spontaneously hypertensive (SH) rats are more susceptible to the development of PM-induced arrhythmia, altered ECG morphology, and reduced HRV than are Wistar Kyoto (WKY) rats, a related strain with normal blood pressure.

**Methods:**

We exposed rats once by nose-only inhalation for 4 hr to residual oil fly ash (ROFA), an emission source particle rich in transition metals, or to air and then sacrificed them 1 or 48 hr later.

**Results:**

ROFA-exposed SH rats developed nonconducted P-wave arrhythmias but no changes in ECG morphology or HRV. We found no ECG effects in ROFA-exposed WKY rats. ROFA-exposed SH rats also had greater pulmonary injury, neutrophil infiltration, and serum C-reactive protein than did ROFA-exposed WKY rats.

**Conclusions:**

These results suggest that cardiac arrhythmias may be an early sensitive indicator of the propensity for PM inhalation to modify cardiovascular function.

Day-to-day variations in airborne particulate matter (PM) associated with air pollution have been linked to increases in cardiovascular morbidity and mortality (Dockery et al. 1993; Pope et al. 1995; Samet et al. 2000; Schwartz et al. 1996), especially in individuals who have preexisting cardiovascular disease (Dockery et al. 2001; Miller et al. 2007; Pope 2000). PM exposure likely exacerbates the cardiac and vascular pathophysiology attendant to hypertension, ischemia, heart failure, diabetes, coronary artery disease, and other dysfunctions of the cardiovascular system. Several mechanisms of PM-induced cardiac dysfunction in humans have been postulated, including autonomic modulation, direct effects of PM constituents on ion channels, myocardial ischemia, and vascular dysfunction related to systemic inflammation (Brook et al. 2003; Schulz et al. 2005; Utell et al. 2002).

The electrocardiogram (ECG) is an important clinical tool that has great prognostic value in the assessment of cardiovascular function. Electrocardiographic recordings in humans have been used to detect abnormal myocardial impulse conduction, cardiac rhythm disturbances, and altered autonomic regulation of heart rate (HR) via the assessment of HR variability (HRV). PM exposure has been linked to changes in ECG morphology, including ST-segment depression (Mills et al. 2007; Pekkanen et al. 2002), altered T-wave amplitude (Henneberger et al. 2005), and QT prolongation (Yue et al. 2007). PM exposure has also been associated with cardiac rhythm changes, including increases in atrial ectopic beats (Sarnat et al. 2006) and ventricular tachyarrhythmias (Riediker et al. 2004). Additionally, fine PM exposures have been associated with low HRV, suggesting an inappropriate increase in sympathetic tone to the heart (Rowan et al. 2007; Zareba et al. 2001). Studies in animal models have yielded a similar spectrum of ECG effects (Campen et al. 2001; Watkinson et al. 1998; Wellenius et al. 2002). However, the utility of any or all of these electrocardiographic end points as sensitive or reproducible toxicologic indicators for targeted pathophysiologic studies of PM and its components in rats is unclear.

Recently, Ostro et al. (2007) demonstrated an association between increased cardiovascular mortality and the fine-PM–associated metals nickel and iron in ambient air in nine California counties. Residual oil fly ash (ROFA), a waste product of fossil fuel combustion from boilers, is rich in the transition metals iron, nickel, and vanadium (Dreher et al. 1997) and, when released as a fugitive particle, is an important contributor to ambient fine PM air pollution (Ghio et al. 2002; Lippmann et al. 2006). Among boiler workers, exposure to ROFA deposits correlated strongly with nocturnal depression of HRV, suggesting a significant influence of transition metals on cardiac function (Cavallari et al. 2008). Kodavanti et al. (2000) demonstrated that hypertensive rats that inhale ROFA develop ST depression during exposure. This sensitivity of hypertensive rats to ST depression suggests that they may also be susceptible to cardiac arrhythmias and changes in HRV. In the present study, we examined the effects of a single acute ROFA inhalation exposure on ECG morphology, HRV, and arrhythmia development in hypertensive and normal rats. Our goal in this study was to establish a reproducible exposure model in sensitive rats to further elucidate the underlying mechanisms of cardiopulmonary responses to PM. We used a well-defined and widely used PM to dissect the various electrocardiagraphic lesions induced with a single inhalation challenge.

## Materials and Methods

### Animals

We obtained adult (12-week-old) male Wistar Kyoto (WKY; background strain for SH rats) and spontaneously hypertensive (SH) rats (SHR/NCrIBR) from Charles River Laboratory (Raleigh, NC). Rats were housed in plastic cages (one per cage), maintained on a 12/12-hr light/dark cycle at approximately 22°C and 50% relative humidity in our Association for Assessment and Accreditation of Laboratory Animal Care–approved facility, and held for a minimum of 1 week before implantation. All protocols were approved by the Institutional Animal Care and Use Committee of the U.S. Environmental Protection Agency. Food (Prolab RMH 3000; PMI Nutrition International, St. Louis, MO) and water were provided *ad libitum*. The animals were treated humanely and with regard for alleviation of suffering. We randomized all rats by weight.

### Surgical implantation of telemeters

We anesthetized animals (WKY-air, *n* = 7; WKY-ROFA, *n* = 8; SH-air, *n* = 8; SH-ROFA, *n* = 8) with an intraperitoneal injection of 1 mL/kg ketamine-xylazine solution (80 mg/mL ketamine HCl, 12 mg/mL xylazine HCl; Sigma Chemical, St. Louis, MO) and implanted them with a biopotential radio-telemetry transmitter [model TA11CTA-F40; Data Sciences International, Inc. (DSI), St. Paul, MN] using aseptic surgical procedures to obtain an ECG signal similar to that of lead II from the standard ECG and to allow measurement of core body temperature (Watkinson et al. 1998). The WKY-air group had one less animal because of postsurgical transmitter malfunction. We allowed animals 2 weeks for recovery from surgery preexposure to ROFA.

### Exposure to ROFA

The ROFA used in this study (Southern Research Institute, Birmingham, AL) was collected as a fugitive stack emission postemission control at a Florida Power & Light plant that burned no. 6 grade residual oil containing 1% sulfur (Hatch et al. 1985) and was the same ROFA sample used by Kodavanti et al (2000). The composition of ROFA included vanadium, nickel, and iron sulfates and has been previously described (Dreher et al. 1997). We selected the target concentration (15 mg/m^3^) for this study to mirror that used by Kodavanti et al. (2000) in an effort to reproduce the effect of ROFA on the ST interval in SH rats, and to assess induction of altered HRV and cardiac arrhythmia. After 2 days of acclimatization, we exposed rats once via nose-only inhalation for 4 hr to 13.4 mg/m^3^ of the PM_2.5_ fraction (mass median aerodynamic diameter, 2.09 μm; geometric SD, 3.37) of ROFA or to filtered air in two separate 24-port nose-only inhalation chambers (Lab Products, Seaford, DE), as previously described (Ledbetter et al. 1998).

### Radiotelemetry data acquisition and analysis

Radiotelemetry allowed continuous monitoring and collection of electrocardiographic data in unanesthetized rats from implantation of the transmitters until sacrifice. We positioned receivers (DataART3.01; DSI) beneath home cages (when the rats were unrestrained) or beneath nose-only inhalation chambers (when rats were restrained in tubes) during exposure. Sixty second segments of ECG waveforms were acquired and saved at 60-min intervals, from surgical recovery through euthanasia; values were obtained sequentially by animal and represent averages of 60 sec of data/animal for each hour. HR was obtained from the ECG waveform. Preexposure data permitted each animal to serve as its own control, and animals exposed to air provided time-paired control data. We obtained preexposure baseline data, that is, one 1-min segment of the ECG waveform/hour, while the animals were unrestrained and in their home cages before nose-only inhalation exposure. We also obtained preexposure baseline data at the same sampling rate while the rats were in the nose-only restraint just before the beginning of exposure. Data were also collected during the nose-only inhalation exposure period once/hour for the duration of the 4-hr exposure period. The rats were then returned to their home cages and postexposure data were collected until euthanasia approximately 48 hr after the beginning of exposure.

### Electrocardiogram

We analyzed each signal using e-MOUSE (Boston, MA), an Internet-based physiologic waveform analysis portal. e-MOUSE incorporates Fourier analyses and linear time-invariant digital filtering of frequencies < 2 Hz and > 100 Hz to minimize environmental signal disturbances, as previously described (Chu et al. 2001). The software uses a peak detection algorithm to find the peak of the R-waves and to calculate HR. We calculated HRV as the mean of the differences between sequential HRs for the complete set of ECG signals. The software plots its interpretation of P, Q, R, S, and T for each beat so that spurious data resulting from unfiltered noise or motion artifacts may be rejected. It then calculates the means of the ECG intervals [i.e., PQ (peak of P-wave to beginning of QRS), PR (peak of P-wave to peak of QRS), QRS, QT, ST, and RR intervals] for each set of waveforms. The QT intervals were rate corrected (QTc) by application of the equation recommended by Mitchell et al. (1998) for use in mice and is applicable to rats. We used ECGAuto software (EMKA Technologies USA, Falls Church, VA) to calculate ST- and T-wave areas. (We calculated ST area as the area below the isoelectric line between the S point of the ECG and the beginning of the T-wave, and T-wave area as the area above the isoelectric line between the beginning of the T-wave and the end of the T-wave.) For each 1-min stream of ECG waveforms, we acquired mean time between successive QRS complex peaks (RR interval), mean HR, and mean HRV-analysis–generated time-domain measures. The time-domain measures included standard deviation of the time between normal-to-normal beats (SDNN), and root mean squared successive differences (RMSSD). HRV analysis was also conducted in the frequency domain using a fast-Fourier transform. The spectral power obtained from this transformation represents the total harmonic variability for the frequency range being analyzed. In this study, we divided the spectrum into low-frequency (LF) and high-frequency (HF) regions. The ratio of these two frequency domains (LF/HF) was calculated as an estimate of the relative balance between sympathetic (LF) and vagal (HF) activity. To account for potential effects of normal circadian rhythm, we quantified both ECG and HRV parameters for time-matched comparisons and compared them at different times of the day during the preexposure and postexposure periods while rats were unrestrained in their home cages. We analyzed only some of the total data collected during the monitoring period: morning (1000 hr), afternoon (1600 hr), and evening (2200 hr). ECG and HRV parameters during exposure were analyzed for baseline (recordings while in nose-only restraint immediately before the beginning of exposure) and for hours 1–4 (constituting the entire exposure period between 0830 hr and 1230 hr).

**Figure 1:**
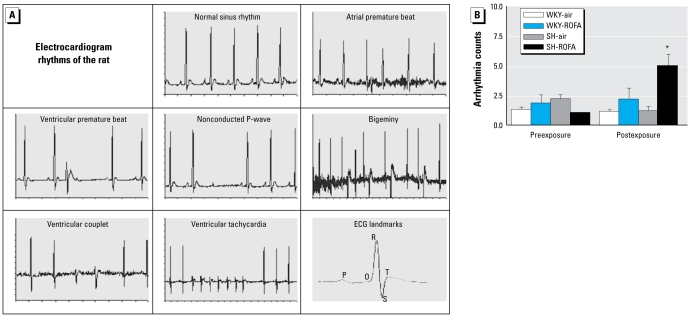
Increased cardiac arrhythmias after ROFA inhalation in SH rats. (*A*) Sample normal and abnormal cardiac rhythms in SH and WKY rats. (*B*) Total nonconducted P-wave arrhythmias during corresponding 24-hr periods pre- and postexposure to ROFA or filtered air: mean number of arrhythmias ± SE (*n* = 7 or 8). The number of arrhythmias in postexposure SH-ROFA rats was significantly greater than in preexposure SH-ROFA rats, postexposure SH-air rats, and postexposure WKY-ROFA rats.*p< 0.05.

ECGAuto software (EMKA Technologies) was used to visualize individual ECG signals for identification and enumeration of specific cardiac arrhythmia events. We used the Lambeth conventions (Walker et al. 1988) as guidelines for the identification of cardiac arrhythmic events in rats. Arrhythmias were identified as nonconducted P-waves, atrial and ventricular premature beats, ventricular bigeminy, ventricular couplets, or ventricular tachycardia. Figure 1A lists examples of these arrhythmias found in the rats used in this study. Arrhythmias are generally infrequent, and were therefore quantified and totaled over a 24-hr period (24 segments of 1 min each) preexposure (corresponding to the same times assessed postexposure), during the 4-hr exposure period (four segments of 1 min each), or during the 24-hr period (24 segments of 1 min each) beginning immediately postexposure.

### Necropsy, blood collection, and bronchoalveolar lavage

We deeply anesthetized rats with an intraperitoneal injection of Euthasol (200 mg/kg sodium pentobarbital, 25 mg/kg phenytoin; Virbac Animal Health, Ft. Worth, TX). Blood samples were collected from the abdominal aorta and renal artery upon exsanguinaton. We cannulated the trachea and lavaged the lungs with a total volume of 35 mL/kg of Ca^2+^/Mg^2+^free, phenol red–free-Dulbecco’s phosphate-buffered saline (SAFC Biosciences, Lenexa, MD) in two equal aliquots. Cytospins and cell differentials on bronchoalveolar lavage (BAL) cell samples; assays for total protein (Coomassie Plus protein reagent; Pierce, Rockford, IL), albumin (Diasorin, Stillwater, MN), lactate dehydrogenase (LDH; ermo DMA, Louisville, CO), and *N*-acetyl-β - -glucosaminidase (Roche Diagnostics, Mannheim, Germany) on BAL supernatants; and serum C-reactive protein (CRP; kit from Diasorin; standard from Kamiya Biomedical Co., Seattle, WA) were conducted as previously described (Farraj et al. 2006).

### Histopathology

We immersed each heart in a large volume of 10% acetate-buffered formalin. After fixation, hearts were trimmed and then processed to paraffin blocks, sectioned at a thickness of 5 μm, placed on glass slides, and stained with hematoxylin and eosin or Barbeito-Lopez trichrome stain for routine diagnosis of myocardial degeneration or necrosis (Milei and Bolomo 1983). Histopathologic changes were assessed by the study pathologist (A. Nyska, Sackler Faculty of Medicine, Tel Aviv University, Tel Aviv, Israel).

### Statistics

We performed statistical analyses for all data in this study using SAS software, version 9.1.3 (SAS Institute, Inc., Cary, NC). PROC MIXED and PROC GLIMMIX procedures were used to analyze all ECG- and HRV-generated data. We used a linear mixed model with restricted maximum-likelihood estimation analysis (SAS) and least-square means post hoc test to determine statistical differences for all data. The arrhythmia data, however, were not normally distributed, so for these we used PROC GENMOD for count data using Poisson regression. All arrhythmia values had one added to them to prevent zero values and to allow model convergence. All the biochemical and differential data were analyzed using a one-, two-, or three-way analysis of variance (ANOVA) model examining the main effects of each model as well as the interactive effects of two- and three-way ANOVA models. We considered *p* < 0.05 statistically significant. A significant interaction in biochemical or differential data resulted in pairwise comparisons performed using Tukey’s post hoc test.

## Results

### HR and HRV

We found no significant differences in HR among any of the groups pre- or postexposure (Table 1) or during exposure (data not shown). There were no significant differences in SDNN, RMSSD, LF, HF, or LF/HF among any of the groups pre- or postexposure (Table 1) or during exposure (data not shown).

### ECG interval duration and morphology

We found no significant differences among any of the groups pre- or postexposure in any of the ECG intervals (Table 2; data for PQ and ST intervals were not statistically signifcantly different and are not shown) or in ST- and T-wave areas (Table 2).

### Cardiac arrhythmia events

Both strains of rats had random and infrequent arrhythmias preexposure, including atrial and ventricular premature beats. We observed very few instances of bigeminy, ventricular couplets, or ventricular tachycardia in either strain pre- or postexposure. There were very few arrhythmias of any type evident during the 4-hr exposure period in any of the rats (data not shown). ROFA exposure caused a 400% increase (*p* < 0.05) in the episodes of nonconducted P-waves in SH rats postexposure (24-hr period) compared with the number of events in the same rats preexposure (time-matched; Figure 1B). The ROFA-induced increase in nonconducted P-waves in SH rats was also > 327% than the average level in air-exposed SH rats postexposure (*p* < 0.05). In addition, the ROFA-induced increase in nonconducted P-waves in SH rats was > 133% than the average level in WKY rats exposed to ROFA postexposure (*p* < 0.05). Most of the nonconducted P-waves took place in the first 12 hr postexposure to ROFA in the SH rats. Mean postexposure counts (± SE) for atrial premature beats were as follows: WKY-air, 1.14 ± 0.14; WKY-ROFA, 2.14 ± 0.40; SH-air, 1.17 ± 0.17; SH-ROFA, 1.57 ± 0.57. Mean postexposure counts for ventricular premature beats were as follows: WKY-air, 1.71 ± 0.17; WKY-ROFA,1.43 ± 0.20; SH-air, 2.0 ± 0.52; SH-ROFA, 1.86 ± 0.46. However, we found no statistically significant exposure- or time-related differences in either type of premature beat (*p*> 0.05).

### Inflammatory cell infiltration into the lung

**Figure 2:**
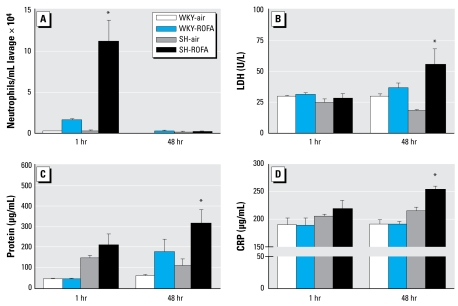
Greater inflammatory response in SH rats after ROFA inhalation. Inflammatory markers in BAL fluid and serum 1 or 48 hr postexposure to ROFA or filtered air. (*A*) BAL neutrophils. (*B*) BAL LDH. (*C*) BAL protein. (*D*) Serum CRP. Values represent mean ± SE (*n* = 7 or 8). *p < 0.05 compared with SH-air and WKY-ROFA rats.

ROFA-exposed SH rats had significantly greater BAL neutrophils than did air-exposed SH rats (6,376% difference; *p* < 0.05) or ROFA-exposed WKY rats (659% difference; *p* < 0.05) 1 hr postexposure (Figure 2A). We found no significant difference in BAL neutrophils between air- and ROFA-exposed WKY rats, or in BAL macrophages between ROFA-exposed SH and WKY rats, at 1 hr postexposure (data not shown). There was no significant difference among any of the treatment groups in macrophages and neutrophils 48 hr postexposure, suggesting that the inflammatory influx into the lung may peak soon after exposure.

### Markers of injury and edema in lung

ROFA-exposed SH rats had significantly greater BAL LDH levels than did air-exposed SH rats (211% difference; *p* < 0.05) or ROFA-exposed WKY rats (54% difference; *p* < 0.05) 48 hr postexposure (Figure 2B). We found no significant differences in any of the exposure groups in BAL LDH levels 1 hr postexposure. ROFA exposure did not significantly increase BAL LDH levels in WKY rats 48 hr postexposure.

ROFA-exposed SH rats had significantly greater BAL protein levels 1 hr postexposure (369% difference; *p* < 0.05) than did ROFA-exposed WKY rats (Figure 2C). In addition, ROFA-exposed SH rats had significantly greater BAL protein levels 48 hr postexposure than did air-exposed SH rats (186% differences; *p* < 0.05) or ROFA-exposed WKY rats (78% difference; *p* < 0.05) 48 hr postexposure. BAL albumin levels were similar to protein levels (data not shown).

We found no significant effect of ROFA exposure in either strain relative to air controls 1 hr postexposure in BAL albumin or protein levels. Unlike the inflammatory influx, injury to the lung required more time to develop.

### Serum CRP

SH rats exposed to ROFA had significantly higher CRP levels than did air-exposed SH rats (18% difference; *p* < 0.05) or ROFA-exposed WKY rats (32% difference; *p* < 0.05) 48 hr postexposure (Figure 2D). We found no significant differences among any of the treatment groups relative to their respective air-exposed controls in serum CRP levels 1 hr postexposure. There was no significant difference between WKY rats exposed to ROFA and WKY rats exposed to air 48 hr postexposure. Like the markers of injury in the lung, elevation in serum CRP required more time to develop than did the inflammatory influx into the lung.

### Heart weight

Mean heart-to-body weight ratios of control rats exposed to air 48 hr postexposure were, for WKY, 3.2 ± 0.2 mg/g, and for SH, 3.53 ± 0.07 mg/g. Thus, SH rats had a > 10.3% heart-to-body weight ratio than did WKY rats, which was statistically significant (*p* < 0.05). We found no statistically significant effect of ROFA inhalation on heart-to-body-weight ratios in either strain.

### Heart histopathology and temperature

There were no PM-exposure–related or strain effects on cardiac tissue morphology or body temperature (data not shown).

## Discussion

In the present study, cardiac arrhythmias were more sensitive indicators of the effects of acute oil fly ash inhalation than were changes in ECG morphology and HRV in hypertensive rats. ROFA inhalation increased the frequency of nonconducted P-waves in hypertensive but not in normal rats during the first 24 hr postexposure. Most of these arrhythmic events occurred during the first 12 hr after ROFA exposure. The nonconducted P-waves in the ROFA-exposed SH rats in this study were preceded by unchanging PR intervals, and thus were similar to Mobitz type II second-degree atrioventricular block observed in humans, which is usually associated with a lesion in the bundle branch conduction pathway (Scheidt 1986). It is unclear, however, whether such lesions exist in rats and whether a single ROFA exposure would be sufficient to elicit such injury. Additionally, the role of autonomic, electrolyte, or other influences in the generation of these cardiac events is uncertain, so the etiology of the nonconducted P-waves that were present in ROFA-exposed SH rats needs to be further studied. Nevertheless, these abnormal rat rhythms are in line with multiple studies that have linked fine PM exposure with the development of cardiac arrhythmias. Fine PM exposure has also been associated with increases in ventricular and supraventricular tachycardia in patients with coronary heart disease (Berger et al. 2006; Dockery et al. 2005), atrial premature beats in patrol officers (Riediker et al. 2004), and atrial and ventricular ectopy in elderly subjects (Sarnat et al. 2006). Analogously, ROFA or its transition metals have been linked to the development of cardiac arrhythmia in rats (Campen et al. 2001; Watkinson et al. 1998; Wellenius et al. 2002). Collectively, these studies suggest that cardiac arrhythmias may be sensitive and quantifiable indicators of the cardiovascular effects of PM in susceptible individuals.

ROFA exposure in both strains of rats failed to cause a decrement in HRV, suggesting that HRV parameters are not as sensitive as cardiac arrhythmias to the effects of acute ROFA inhalation in this model. HRV is the degree of difference in the interbeat intervals of successive heartbeats and is an indicator of the balance between the sympathetic and parasympathetic arms of the autonomic nervous system (Rowan et al. 2007). Low HRV, reflecting inappropriately increased sympathetic tone (Rowan et al. 2007), is associated with an increased risk of cardiac arrhythmia (Corey et al. 2006) and an increased mortality rate in people with heart disease (Bigger et al. 1993; Fauchier et al. 2004). An association between high ambient PM and low HRV has been observed in several different studies in both healthy and diseased individuals (Chuang et al. 2005; Muggenburg et al. 2000; Schulz et al. 2005; Vallejo et al. 2006). Other investigators, however, have reported increases in HRV with exposure to vanadium in ambient PM (Magari et al. 2002) and fine PM mass itself (Timonen et al. 2006). The data in this study and others suggest that the effects of PM pollution on HRV and the prognostic value of HRV in air pollution studies are uncertain.

ROFA exposure in both strains of rats failed to cause any changes in ECG interval duration, including RR and QT intervals, suggesting that such end points may not be sensitive to acute PM exposure in this model of hypertension. In addition to interval time, the morphology of the ECG waveform can also provide information about cardiac physiology. Air pollution exposure has been associated with changes in ventricular repolarization as indicated by altered T-wave morphology, or T-wave alternans, in patients with ischemic heart disease (Henneberger et al. 2005). Changes in repolarization may trigger arrhythmogenesis and thus have been used to identify patients at risk for cardiac death (Henneberger et al. 2005). The ST segment of the ECG is most sensitive to drugs or disease (DSI 2002), and ST depression is temporally associated with myocardial ischemia (Scheidt 1986). Ischemic episodes are negative prognostic indicators that point to an increase in the probability of future cardiac events, including myocardial infarction (Tzivoni et al. 1988). Traffic-related PM has been linked with ST-segment depression in elderly patients (Gold et al. 2005) and patients with coronary heart disease (Pekkanen et al. 2002). Kodavanti et al. (2000) performed a qualitative assessment of ECG signals derived from SH rats and found that ROFA caused an ST depression during the first two of three inhalation exposures (Kodavanti et al. 2000). Rats lack an equivalent human ST-segment because of the fast rate at which their ventricular myocytes repolarize (DSI 2002). Notwithstanding this difference, perturbations that result in ST-segment changes in species with ST segments produce a similar shift in the corresponding portion of the ECG in rats (DSI 2002). In our study, we found no significant differences in ST- or T-wave areas among any of the exposure groups or strains, suggesting no evidence of ST depression or altered ventricular repolarization. The inability to clearly reproduce the findings of Kodavanti et al. (2000) may be related to subtle differences between the studies (duration or concentration). On the other hand, these findings suggest that such a change may not be consistent because of confounders affecting sensitivity to challenge at rest. An analysis of ST-segment changes may be more appropriate when HR is elevated, as in exercise (as found in humans; Pekkanen et al. 2002; Mills et al. 2007), because oxygen demand is elevated, thus increasing the likelihood of ischemic episodes, which in turn may be reflected in ST changes in the ECG (Scheidt 1986). Such tests may be adaptable to animal bioassays to better assess the effects of PM or other pollutants on cardiac ischemic changes or changes in repolarization reflected in the ECG.

The inhalation of fine PM has been linked to both pulmonary and systemic inflammation (Brook et al. 2003; Utell et al. 2002), including increases in the circulating acute-phase marker CRP and increases in indicators of endothelial dysfunction and injury in the circulation (Calderon-Garciduenas et al. 2008). Episodes of cardiac dysfunction, including cardiac arrhythmias, may result from systemic inflammation via altered coagulative processes, destabilization of arterial plaques, and/or exacerbation of atherosclerotic plaque formation (Brook et al. 2003). Elevated CRP paralleling ECG changes, including QT prolongation and decreased T-wave amplitude, has also been reported in coronary artery disease patients exposed to ambient fine PM (Yue et al. 2007). Furthermore, systemic inflammation may actually trigger cardiac arrhythmia as evidenced by the finding that elevated serum CRP was predictive of the recurrence rate of atrial fibrillation, another type of cardiac arrhythmia (Hatzinikolaou-Kotsakou et al. 2006). SH rats exposed to ROFA in the present study had significantly higher pulmonary influx of neutrophils, markers of pulmonary edema and injury, including LDH, protein, and albumin, and higher serum CRP levels than did similarly exposed WKY rats with normal blood pressure; these pulmonary responses were quite similar to the findings of Kodavanti et al. (2000) after three exposures. The strong, strain-dependent inflammatory response in SH rats exposed to ROFA in this study may have contributed to the development of the nonconducted P-wave arrhythmias.

The incidence of nonconducted P-wave arrhythmias increased only in SH rats exposed to ROFA, suggesting that hypertension conditions the heart, creating a substrate that increases the susceptibility to PM-induced arrhythmia. Although we did not measure blood pressure in this study, SH rats have significantly higher mean arterial pressure relative to their related background strain, the WKY rats (Yamori et al. 1979). Hypertension over time leads to hypertrophy of the left ventricle (American Heart Association 2008), a condition present in the SH rats of this study as evidenced by their higher heart-to-body weight ratio compared with WKY rats. Several remodeling events take place in the hypertrophying heart that may heighten myocardial sensitivity to the effects of PM exposure, including *a* ) prolonged QT intervals and action potential duration resulting from changes in calcium ion channel density, *b*) aberrant expression of proteins/currents specific to the sinoatrial node, suggesting that the ventricles can acquire automaticity, and *c*) an increase in the density of connexin 40 (a gap-junction protein involved in cell-to-cell electrical coupling), which is usually confined to the atria and has greater conductance (Swynghedauw 1999). That SH rats undergo left ventricular hypertrophy is well documented (Yamori et al. 1979), suggesting that the SH rats in this study may have undergone some of these remodeling events during the course of disease progression that predisposed them to the development of ROFA-induced nonconducted P-wave arrhythmias. The absence of any clear histologic distinction between SH and WKY rat hearts in this study suggests that the differences may be at the ultrastructural and molecular levels. Nevertheless, these findings clearly point to a compromising effect of hypertension on the responsiveness to ROFA in SH rats.

In summary, compared with HRV and ECG morphology in this rat model of hypertension, cardiac arrhythmias, particularly nonconducted P-waves, were the most sensitive indicators of the adverse cardiovascular effects of ROFA inhalation, suggesting that cardiac arrhythmias may have predictive value in the assessments of the effects of air pollution. This model may in turn be used to compare the capacity of other PM samples to elicit cardiac arrhythmias in the context of hypertension, potentially facilitating comparisons of the cardiovascular toxicity of PM from different air sheds. In addition, PM-induced cardiac arrhythmias found in this model may help elucidate the mechanism of action. One additional mechanism that requires further study involves direct injury to cardiac myocytes resulting from the translocation of metals to the heart after inhalation. ROFA metals, including nickel and vanadium, leach into the blood from the lung and enter other organs, including the heart (Wallenborn et al. 2007), and thus may have had a direct role in the development of nonconducted P-wave arrhythmias in this study. This study also demonstrated that normal animals have very minimal responses to very high acute PM exposure, with only a mild inflammatory response and no evidence of cardiac arrhythmia. Healthy humans or animals may thus be more resistant to the effects of air pollution compared with diseased cohorts, suggesting that the effects of air pollution should more appropriately be assessed in the context of preexisting disease. Factors that may influence the sensitivity of ECG end points that we did not address in this study include repeated or chronic exposures to ROFA, exposure to lower concentrations of ROFA, exposure to other PM samples, or the exposure of animals with other cardiovascular diseases. Taken together, the data suggest, however, that cardiac arrhythmia may be an early sensitive ECG indicator of the propensity for PM inhalation to modify cardiovascular function and trigger acute cardiac events.

## Figures and Tables

**Table 1:** HR and HRV pre- and postexposure

Time	Strain	Exposure	HR (bpm)	SDNN (bpm)	RMSSD (msec)	LF (msec^2^ )	HF (msec^2^ )	LF/HF
Morning baseline (preexposure)	WKY	Air	300.3 ± 12	11.31 ± 1.9	4.07 ± 1.1	7.05 ± 2.9	1.31 ± 0.3	11.93 ± 4.6
		ROFA	313.9 ± 12	16.11 ± 1.9	5.84 ± 1.1	11.49 ± 2.9	1.91 ± 0.3	7.35 ± 4.6
	SH	Air	311.5 ± 13	12.17 ± 2.1	3.29 ± 1.1	8.47 ± 3.1	1.11 ± 0.4	13.82 ± 5.0
		ROFA	287.5 ± 7.0	16.8 ± 3.7	3.58 ± 1.0	9.44 ± 2.7	1.39 ± 0.3	9.24 ± 4.3

Morning (postexposure)	WKY	Air	270.6 ± 7.8	13.09 ± 3.9	6.43 ± 1.1	20.75 ± 7.9	4.74 ± 2.8	5.52 ± 3.1
		ROFA	288.0 ± 8.0	12.79 ± 3.9	5.83 ± 1.1	13.19 ± 7.9	4.54 ± 2.8	4.93 ± 3.1
	SH	Air	273.8 ± 8.4	10.32 ± 4.2	3.65 ± 1.2	7.31 ± 8.6	1.59 ± 3.1	7.06 ± 3.3
		ROFA	287.5 ± 7.3	16.8 ± 3.7	5.17 ± 1.0	22.03 ± 7.4	5.75 ± 2.7	7.34 ± 2.9

Afternoon baseline (preexposure)	WKY	Air	306.6 ± 38	19.57 ± 19	4.03 ± 3.2	9.04 ± 7.7	1.26 ± 16	9.30 ± 3.1
		ROFA	385.7 ± 38	55.6 ± 22	10.50 ± 3.2	18.98 ± 7.7	29.67 ± 16	3.87 ± 3.1
	SH	Air	300.0 ± 41	22.10 ± 24	7.45 ± 3.4	21.97 ± 8.3	20.66 ± 17	10.14 ± 3.3
		ROFA	298.3 ± 36	9.86 ± 20	4.64 ± 0.7	10.70 ± 7.2	1.78 ± 15	6.15 ± 2.9

Afternoon (postexposure)	WKY	Air	281.3 ± 12	16.13 ± 3.4	4.56 ± 0.8	15.95 ± 5.5	2.06 ± 1.3	8.30 ± 5.8
		ROFA	307.6 ± 12	17.93 ± 3.4	4.74 ± 0.8	15.15 ± 5.5	1.82 ± 1.3	10.74 ± 5.7
	SH	Air	305.3 ± 13	12.33 ± 3.6	3.52 ± 0.8	7.66 ± 6.0	1.41 ± 1.4	10.56 ± 6.2
		ROFA	354.4 ± 11	20.15 ± 3.1	4.64 ± 0.7	12.18 ± 5.2	3.42 ± 1.2	15.83 ± 5.4

Evening baseline (preexposure)	WKY	Air	327.1 ± 10	15.80 ± 4.4	5.23 ± 0.7	8.79 ± 12	2.94 ± 1.0	3.28 ± 5.73
		ROFA	344.9 ± 10	17.34 ± 4.4	3.47 ± 0.7	26.64 ± 12	2.25 ± 1.0	12.39 ± 5.7
	SH	Air	319.2 ± 11	14.18 ± 4.7	2.85 ± 0.7	17.77 ± 13	0.72 ± 1.1	21.41 ± 6.2
		ROFA	335.8 ± 13	13.00 ± 4.1	4.44 ± 0.6	11.1 ± 11	2.36 ± 0.9	7.74 ± 5.4

Evening (postexposure)	WKY	Air	333.0 ± 14	12.24 ± 3.5	4.16 ± 2.2	6.37 ± 10	1.63 ± 1.0	4.07 ± 9.9
		ROFA	335.6 ± 14	17.49 ± 3.5	3.66 ± 2.2	19.93 ± 10	1.70 ± 1.0	29.71 ± 9.9
	SH	Air	309.3 ± 15	17.00 ± 3.7	3.51 ± 2.3	25.87 ± 11	1.28 ± 1.1	21.33 ± 11
		ROFA	335.8 ± 13	16.53 ± 3.2	8.06 ± 2.0	19.18 ± 9.4	2.96 ± 0.9	8.5 ± 9.3

**Table 2:** ECG interval durations pre- and postexposure

Time	Strain	Exposure	PR (msec)	QRS (msec)	QTc (msec)	RR (msec)	ST area	T-wave area
Morning baseline (preexposure)	WKY	Air	47.86 ± 1.3	18.23 ± 1.7	71.06 ± 2.2	201.0 ± 7.0	0.51 ± 0.24	0.76 ± 0.25
		ROFA	47.76 ± 1.3	16.73 ± 1.7	70.19 ± 3.0	194.3 ± 7.0	0.38 ± 0.13	1.39 ± 0.41
	SH	Air	45.98 ± 1.4	17.83 ± 1.9	68.50 ± 2.1	195.3 ± 7.0	0.92 ± 0.23	0.64 ± 0.55
		ROFA	44.50 ± 1.2	18.29 ± 1.6	70.89 ± 1.7	209.4 ± 6.4	0.66 ± 0.10	0.43 ± 0.26

Morning (postexposure)	WKY	Air	50.97 ± 1.9	16.97 ± 1.5	70.92 ± 1.8	223.4 ± 6.0	0.64 ± 0.16	0.87 ± 0.33
		ROFA	47.04 ± 1.3	17.56 ± 1.5	71.37 ± 2.5	209.7 ± 6.0	0.40 ± 0.13	1.54 ± 0.46
	SH	Air	45.43 ± 2.0	21.47 ± 1.6	76.94 ± 1.4	221.5 ± 6.5	0.96 ± 0.28	0.64 ± 0.26
		ROFA	46.54 ± 1.8	20.70 ± 1.4	73.63 ± 1.9	209.9 ± 5.6	0.65 ±.0.24	0.95 ± 0.39

Afternoon baseline (preexposure)	WKY	Air	49.31 ± 3.2	19.07 ± 1.5	70.73 ± 2.2	198.4 ± 11	0.36 ± 0.12	0.46 ± 0.11
		ROFA	44.36 ± 3.2	15.86 ± 1.5	65.66 ± 4.0	178.5 ± 11	0.62 ± 0.12	1.12 ± 0.43
	SH	Air	45.67 ± 3.4	17.02 ± 1.7	67.00 ± 1.8	203.5 ± 11	0.69 ± 0.27	1.04 ± 0.48
		ROFA	49.00 ± 3.0	19.61 ± 1.4	68.89 ± 1.4	202.4 ± 10	0.84 ± 0.16	0.41 ± 0.19

Afternoon (postexposure)	WKY	Air	47.39 ± 1.4	18.63 ± 1.5	71.82 ± 2.6	215.2 ± 7.0	0.44 ± 0.11	0.54 ± 0.17
		ROFA	46.16 ± 1.4	17.76 ±1.5	69.11 ± 3.0	197.6 ± 7.0	0.41 ± 0.16	1.70 ± 0.37
	SH	Air	43.68 ± 1.5	18.50 ± 1.6	68.51 ± 2.2	199.0 ± 7.0	0.81 ± 0.16	0.69 ± 0.24
		ROFA	42.98 ± 1.3	17.71 ± 1.4	65.95 ± 1.5	171.2 ± 6.0	0.47 ± 0.11	1.41 ± 0.32

Evening baseline (preexposure)	WKY	Air	46.97 ± 1.1	16.40 ± 1.1	66.57 ± 1.2	184.9 ± 6.1	0.46 ± 0.09	0.70 ± 0.22
		ROFA	45.51 ± 1.1	16.21 ± 1.1	67.07 ± 1.6	176.3 ± 6.1	0.39 ± 0.12	1.72 ± 0.70
	SH	Air	43.37 ± 1.2	18.73 ± 1.2	67.28 ± 1.6	189.7 ± 6.6	1.12 ± 0.21	0.66 ± 0.30
		ROFA	43.95 ± 1.0	18.56 ± 1.0	66.46 ± 1.3	194.8 ± 5.7	0.93 ± 0.21	0.39 ± 0.20

Evening (postexposure)	WKY	Air	45.87 ± 1.4	16.77 ± 1.4	65.05 ± 2.1	182.7 ± 7.3	0.43 ± 0.09	0.47 ± 0.18
		ROFA	41.67 ± 1.4	15.39 ± 1.4	67.31 ± 2.7	181.1 ± 7.3	0.43 ± 0.09	1.28 ± 0.49
	SH	Air	44.68 ± 1.5	19.75 ± 1.5	70.62 ± 2.1	195.6 ± 7.9	0.80 ± 0.24	1.08 ± 0.23
		ROFA	44.08 ± 1.3	19.06 ± 1.3	69.11 ± 2.9	181.7 ± 6.9	0.71 ± 0.16	1.02 ± 0.42
